# Adhesive ligand tether length affects the size and length of focal adhesions and influences cell spreading and attachment

**DOI:** 10.1038/srep34334

**Published:** 2016-09-30

**Authors:** Simon J. Attwood, Ernesto Cortes, Alexander William M. Haining, Benjamin Robinson, Danyang Li, Julien Gautrot, Armando del Río Hernández

**Affiliations:** 1 Cellular and Molecular Biomechanical laboratory, Department of Bioengineering, Imperial College London, United Kingdom; 2 Institute of Bioengineering School of Engineering and Materials Science, Queen Mary, University of London, Mile End Road, London, E1 4NS, UK; 3 School of Engineering and Materials Science, Queen Mary, University of London, Mile End Road, London, E1 4NS, UK

## Abstract

Cells are known to respond to physical cues from their microenvironment such as matrix rigidity. Discrete adhesive ligands within flexible strands of fibronectin connect cell surface integrins to the broader extracellular matrix and are thought to mediate mechanosensing through the cytoskeleton-integrin-ECM linkage. We set out to determine if adhesive ligand tether length is another physical cue that cells can sense. Substrates were covalently modified with adhesive arginylglycylaspartic acid (RGD) ligands coupled with short (9.5 nm), medium (38.2 nm) and long (318 nm) length inert polyethylene glycol tethers. The size and length of focal adhesions of human foreskin fibroblasts gradually decreased from short to long tethers. Furthermore, we found cell adhesion varies in a linker length dependent manner with a remarkable 75% reduction in the density of cells on the surface and a 50% reduction in cell area between the shortest and longest linkers. We also report the interplay between RGD ligand concentration and tether length in determining cellular spread area. Our findings show that without varying substrate rigidity or ligand density, tether length alone can modulate cellular behaviour.

It is known that cells are able to sense the rigidity of their microenvironment as illustrated by their differential behaviour when cultured on soft versus stiff substrates. Cell differentiation^1^,^2^,^3^, rate of DNA synthesis^4^, apoptosis^4^, traction forces^4^, motility^5^,^6^,^7^,^8^ and spread area^2^,^4^,^5^,^7^,^8^,^9^,^10^,^11^ have all been shown to be modulated by changes in substrate rigidity. It has also been demonstrated that transformed cancer cells respond differently to substrate stiffness compared to normal cells^4^,^12^,^13^,^14^. Furthermore, the density of extracellular matrix (ECM) proteins such as collagen and fibronectin play a major role in determining cell behaviour^9^,^15^,^16^,^17^.

In order for cells to sense the rigidity of the ECM, they must first form linkages with ECM proteins via transmembrane integrin receptors^18^. Fibronectin is an ECM protein that connects to cell surface integrins via a discrete section along its length containing the adhesive RGD ligand^19^,^20^. With the rest of the fibronectin protein playing a passive role in adhesion, a picture emerges of cells tethered to the matrix via thin and flexible strands of varying length. Within this scenario, we considered the possibility that cells are receptive not only to ECM rigidity and adhesive ligand density but also to the length of the local tether to which the ligand attaches to the broader ECM microenvironment.

It was previously argued that, on poly(acrylamide) and poly(dimethylsiloxane) substrates functionalized with ECM proteins, cellular responses due to modulation of substrate stiffness were due to concomitantly modifying the fibronectin or collagen tether density, which resulted in substantial changes in nanoscale mechanical properties^11^. Subsequently, it was found that varying the apparent porosity of poly(acrylamide) gels in order to control such tethering density did not result in changes in cell behaviour^17^. This indicates that apparent porosity alone is not sufficient to account for the observations made; however, the approach developed did not permit the direct measurement of the density of tethers between the matrix and deposited ECM proteins. Notably, it was not possible to vary the apparent porosity without altering the density of polymer chains in the sample, which is expected to contribute significantly to ECM tethering density. Importantly, the work of Chen and co-workers using micropillars^2^, and that of others^21^,^22^, indicate that cells respond to the mechanical properties of their environment. In these cases, the bulk mechanical properties of the substrates were not varied, but micropillars were used to modulate the flexural moduli perceived by cells. This raises the possibility that cells primarily sense deformation of the matrix, accounting for the observations made on micropillars and for substrates displaying variations in their tethering density. The associated reduction in nanoscale mechanical coupling would result in a decreased ability to deform the matrix at the nanoscale. However, until now no study has provided quantitative evidence that, without modulating substrate stiffness, adhesive-ligand tether length alone provides a strong mechanical signal that is able to modulate cellular behaviour.

## Results

### Functionalization and validation of surfaces with tethers of different lengths

In order to investigate the effect of tether length on cellular behaviour, we covalently coupled RGD peptides to glass surfaces via inert polyethylene glycol tether molecules. By varying the number of repeats of the ethylene glycol subunits, we were able to prepare surfaces with short (9.5 nm), medium (38.2 nm) and long (31.8 nm) length tethers. The three amino acid peptide sequence, RGD, is minimally required for cellular adhesion to surfaces and recapitulates the adhesion properties of the ECM protein fibronectin^19^. A three-step chemistry procedure was used to prepare the surfaces, which was adapted from a previously described protocol^23^. The PEG linkers are inert to cells, therefore allowing us to control the length parameter only, without altering the adhesive properties. The high flexibility and mobility of the PEG molecules also permits the RGD ligand to be presented in the correct orientation for adhesion to occur with the transmembrane integrin receptors. The lengths of the linkers span the approximate range of single native fibronectin molecules^24^. However, this wide range of tether lengths is unlikely to be mimicked in the real physiological environment. In order to facilitate testing the cellular system of mechanotransduction we believe it to be insightful to present an extreme range of length.

**Figure 1:**
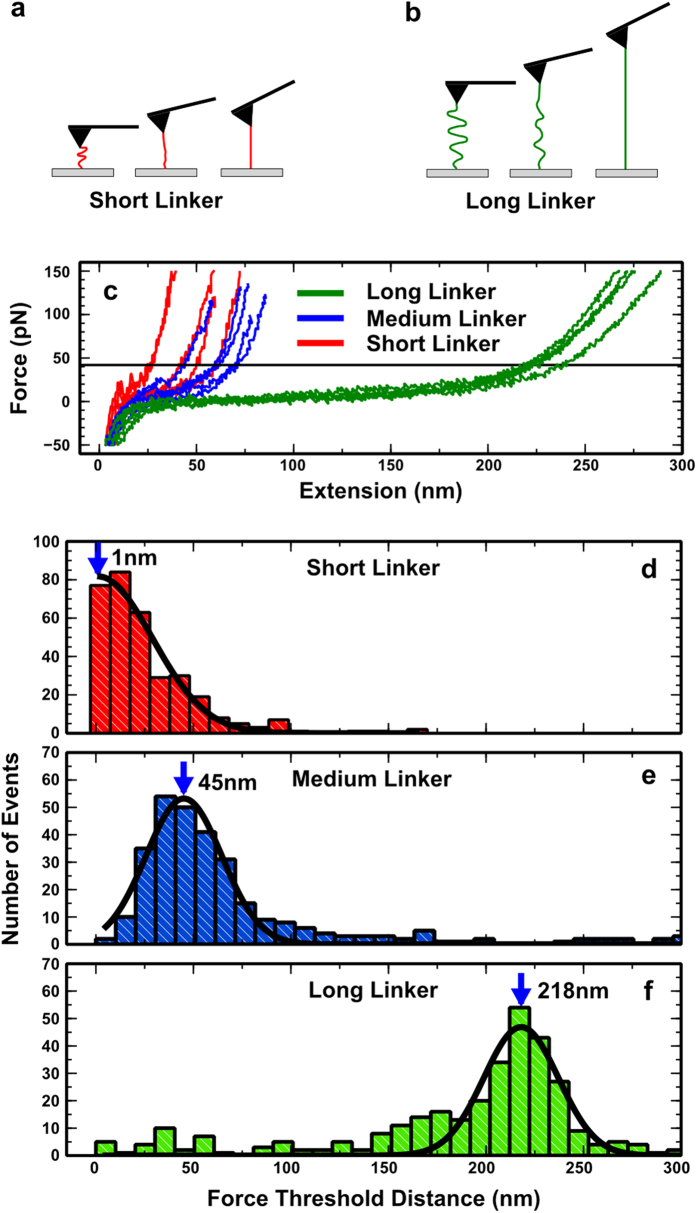
Atomic force spectroscopy used to probe tether length. Schematic illustrating the single molecule approach to probing the short (**a**) and long (**b**) polyethylene glycol (PEG) tethers. As the cantilever is retracted upwards the force due to the tension in the linker causes the lever to be deflected, which is measured as a force. (**c**) Representative force-extension traces for short, medium, and long linkers. The dashed line represents the force required to activate integrins (43pN). Histograms of distances taken to reach the 43pN threshold force for short (**d**), medium (**e**) and long (**f**) length tethers together with Gaussian fits to the data and peak lengths (n = 300 for each).

In order to test the effectiveness of the coupling procedure and to assess the nature of the forces a coupled cell might experience, the surfaces were characterised using Single Molecule Atomic Force Spectroscopy (smAFM) as illustrated in Fig. 1a,b. Using this approach, we measured force-extension profiles for single PEG strands on each of the three surfaces and determined the extension distance at which the tension in the PEG tether reaches the critical 43pN reportedly required to activate integrins^25^,^26^. In Fig. 1c we present representative force-extension traces. The force on a short linker rose quickly above the threshold force whereas for the longest linker there was a long lag period during retraction where only low forces were experienced, after which the force rose steeply above the threshold force. It should be noted that this type of force profile with a varied length lag period leading to a steep force increase is very different to the linear force-extension profiles expected for other substrates such as polyacrylamide gels often used in cell contraction studies. The data suggest that we are therefore able to decouple the stiffness and tether length that the cell encounters. Figure 1d–f show histograms of extension distances taken to reach the threshold force (n = 300 for each) together with Gaussian fits to the data and measured peak values. The extensions at which the threshold force is crossed are 1 nm, 45 nm and 218 nm for the short, medium and long linkers respectively. These correlate well with the assumed lengths of these linkers based on their molecular weight (9.5 nm, 38.2 nm and 318 nm respectively).

### The length of the adhesive linkers determines the size and length of focal adhesion complexes

**Figure 2:**
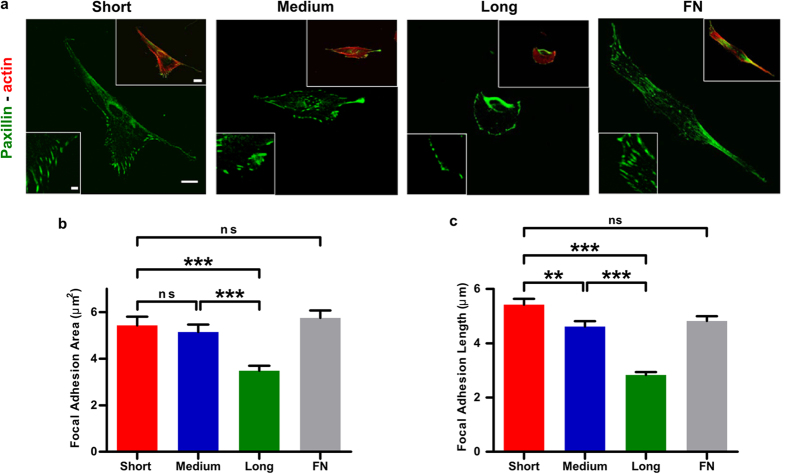
Adhesive ligand tether length affects the size and length of focal adhesions. (**a**) Confocal microscopy images of cells stained for paxillin (green) and F-actin (red) on short, medium and long tethered surfaces and on fibronectin-coated glass (FN). Scale bars are 20 μm in main image and cell inset and 5 μm in FAs inset. (**b**) Quantification of focal adhesion length and area (n > 85 for each condition). Histograms represent mean ± sem.

Focal adhesion complexes regulate the reciprocal mechanical communication between the cell and the ECM and mediate cell adhesion and spreading^27^. To understand how linker length affects cell adhesion and spreading, we characterised focal adhesion complexes of cells plated on surfaces coupled with each of the three linkers. For this, human foreskin fibroblasts were seeded onto these three surfaces in serum-free media. Serum components contain cell adhesive protein fibronectin, thus it is important to exclude this parameter and to ensure any observed cellular behaviour is due only to the RGD-coupled molecules. Using immunofluorescence, we observed that the length and size of the paxillin-containing focal adhesions gradually and significantly decreased two-fold from short to long linkers (Fig. 2b,c). On short linker surfaces, long wedge shaped mature focal adhesions developed, similar to those seen on the fibronectin-coated glass surfaces (Fig. 2a). As the tether length increased, there was progression towards smaller, more circular focal adhesions, which is consistent with less mature adhesions^28^.

Cells plated on all three surfaces (short, medium, and long linkers) organized mature focal adhesions that were generated within minutes, were around 2 μm wide, and range between 3 and 10 μm in length (Fig. 2c)^28^,^29^,^30^. Notably, cells attached to the long linker-coated substrates developed focal adhesions which were within the lower boundaries reported for focal adhesion sizes^28^. Most cells plated on short linker-coated surfaces displayed uniformly oriented focal adhesions (Fig. 2a). Focal adhesions from cells seeded on medium linkers exhibited a more disorganized orientation whilst cells on long linker-coated surfaces formed much smaller and radially oriented adhesions. Moreover, cells attached to short linkers adopted the typical fibroblast-like polarized phenotype with a lamellar front that protruded occasionally to form filopodia. Cell polarization and protrusions decreased in cells plated on medium linkers and were minimal for cells attached to long linkers (Fig. 2a).

### Adhesive ligand tether length affects both cell spread area and cell surface density

**Figure 3:**
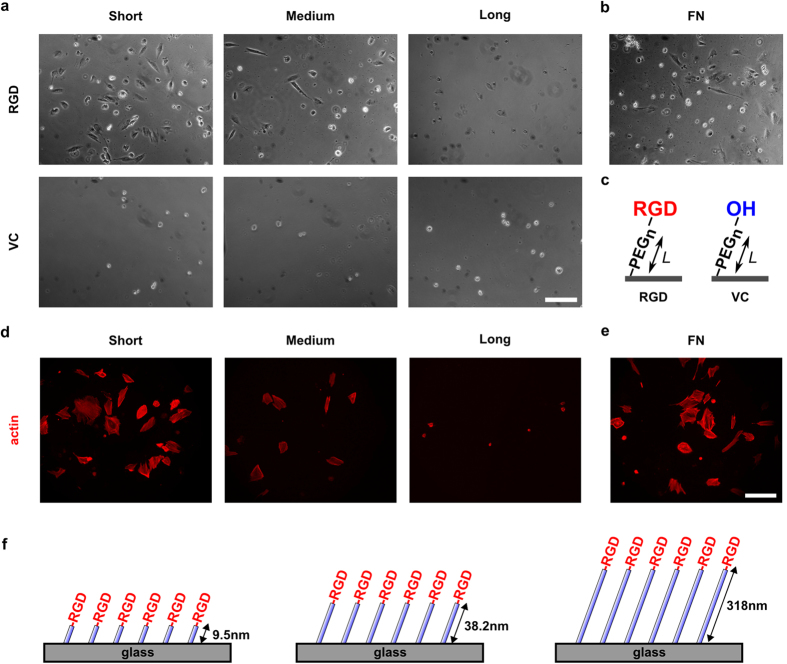
Adhesive ligand tether length affects both cell spread area and cell surface density. (**a**) Upper row: Phase contrast images of cells on RGD-coupled surfaces prepared with short (9.5 nm), medium (38.2 nm) and long (318 nm) polyethylene glycol (PEG) tethers. Cell spread area and cell attachment decrease with increasing tether length; Lower row: Vehicle control (VC) surfaces with short, medium and long length tethers but lacking the RGD adhesive ligand. All cells appear small and rounded. (**b**) Cells on fibronectin-coated glass (FN) exhibit similar cell surface density and spread area compared to cells on RGD surfaces with the shortest tethers. (**c**) Schematic diagram illustrating the coupling procedure for RGD, and the vehicle control. (**d**) Epifluorescence images of cells stained for F-actin (phalloidin) on short, medium and long tethered surface. (**e**) Cells on fibronectin-coated glass (FN) stained for F-actin. Scale bars 200 mm. (**f**) Schematic illustrating the surface structure of the three surfaces.

Consistent with the size and length of focal adhesions, cells seeded on RGD surfaces with the shortest tether were well spread and displayed a greater density of cells attached to the surface, comparable to cells seeded for the same time on glass surfaces with a fibronectin coating (Fig. 3b). This confirmed that the RGD peptide is acting as an effective adhesive ligand for the cells. Additionally, both the spread area and surface density of cells dramatically decreased as the RGD tether length increased. For cells seeded on surfaces that lack the RGD peptide sequence (vehicle control), however, we only observed rounded cells distributed infrequently across the surface (Fig. 3a,b,d–f). This demonstrates the inert nature of the PEG groups to cellular adhesion and also confirms that the cells are specifically responding to the RGD sequence. We also note that the PEG coating should make the glass surface relatively protein resistant.

**Figure 4:**
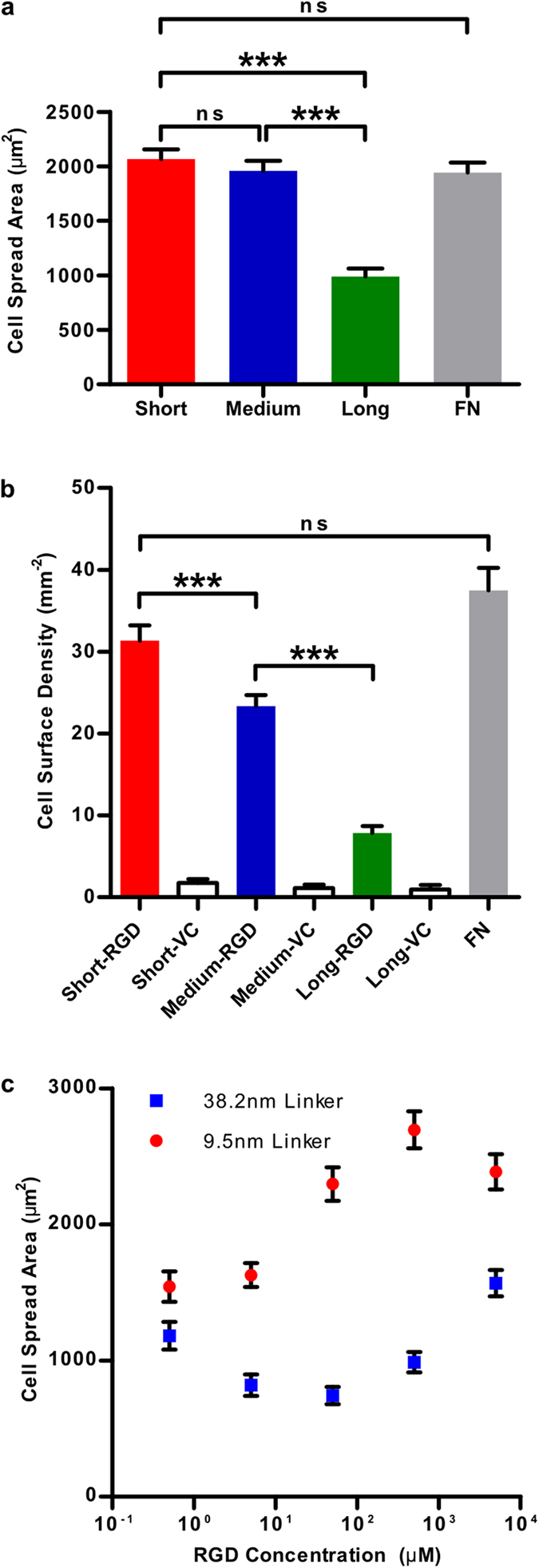
Quantification of cell spreading area and cell density as a function of tether length. (**a**) Cell spread area for surfaces with short (9.5 nm), medium (38.2 nm) and long (318 nm) polyethylene glycol (PEG) tethers as well as fibronectin-coated glass (FN). We observe a reduction of more than 50% in spread area between the shortest and longest linker tethered surfaces. (**b**) Cell surface density (number of cells per unit area) for all three RGD surfaces, vehicle control surfaces (no RGD) and the fibronectin-coated surface. We observe a tether length-dependent decrease in cell surface density (which is a measure of cell adhesion behaviour) with high significance between all three surfaces. There is a 75% decrease in cell surface density between the shortest and longest tethers. On average there is a 92% reduction between RGD and vehicle control surfaces, indicating a high level of specificity. The cell surface density on the fibronectin-coated surface is comparable to that on the shortest RGD-tethered surface. More than 200 cells were measured for each experimental condition. (**c**) Interplay between RGD concentration, cell spread area and RGD tether length. At every concentration we observe the cell spread area to be significantly lower for the medium length linker (38.2 nm) as compared to the short linker (9.5 nm). The greatest difference is observed at 50 μM where we observe a 68% decrease in the spread area from 2300 ± 100 μm2 to 740 ± 60 μm2 between the short and medium length linker respectively.

The spread area and surface density were determined for more than 200 cells across four different samples for each experimental condition. All samples were prepared in parallel for the data presented. As seen in Fig. 4a, the average spread area decreased with increasing tether length: 2070 ± 90 μm^2^ (short tether), 1960 ±90 μm^2^ (medium tether) and 990 ± 73 μm^2^ (long tether). There was a more than 50% reduction in the spread area comparing the shortest to the longest linker tethered surfaces, which is highly statistically significant. Cells on fibronectin coated surfaces had an average spread area of 1940 ± 90 μm^2^, comparable to the cells on the short and medium length tethered surfaces.

Since the surfaces were washed at the 17 hr point and before fixation, the surface density of cells (number of cells per unit area) acts as a measure of cellular adhesion. Cell surface density, and therefore adhesion, varied in a linker length-dependent manner with a high statistical significance between all three surfaces (Fig. 4b). Surface density values for the three surfaces were 31 ± 2 mm^−2^ (short tether), 23 ± 1 mm^−2^ (medium tether) and 7.9 ± 0.9 mm^−2^ (long tether). Remarkably there was a 75% reduction in cell density between samples prepared with the shortest and longest tethers. The surface density of cells on the fibronectin coated surface was 38 ± 3 mm^−2^ which is statistically comparable to the cell density of the shortest tethered sample. The vehicle control samples all exhibited a very low surface density: 1.7 ± 0.5 mm^−2^ (short tether), 1.1 ± 0.4 mm^−2^ (medium tether) and 1.0 ± 0.6 mm^−2^ (short tether) with no statistical difference between them. On average there was a 92% reduction between RGD-coupled surfaces and the vehicle control surfaces, again indicating a highly specific response.

In order to further assess the interplay between RGD ligand density and tether length, we varied the surface density of the RGD ligand by varying the solution concentration of RGD during functionalization of the surfaces over five orders of magnitude. Results for surfaces prepared with the short and medium length tether are shown in Fig. 4c. For the short linker, there was a steady increase in the spread area from 1500 ± 100 μm^2^ to 2700 ± 100 μm^2^ whilst varying the RGD concentration from 0.5 μM to 0.5 mM. At the highest concentration of 5 mM, there was a slight decrease in the spread area to 2400 ± 100 μm^2^. This saturation effect has been seen previously with varying collagen surface density^9^. For the medium length linker, it can be seen that for any given concentration the spread area is significantly decreased when compared to the shorter length linker. The greatest difference was observed at 5 μM where there was a 68% decrease in the spread area from 2300 ± 100 μm^2^ to 740 ± 60 μm^2^ between the short and medium length linker respectively. It is clear from these results that there is interplay between tether length and RGD density. The largest spread area can be achieved by combining the shortest tether with the highest density of RGD (no greater than 0.5 mM) and similarly the lowest spread area can be achieved with the longest tether combined with the lowest RGD density. We see almost the same spread area for the short linker at the lowest concentration as we do for the medium length linker at the highest concentration (1500 ± 100 μm^2^, 0.5 μM versus 1600 ± 100 μm^2^, 5 mM).

### Impact of modest changes in ligand density vs. tether length on focal adhesions and cell density

**Figure 5:**
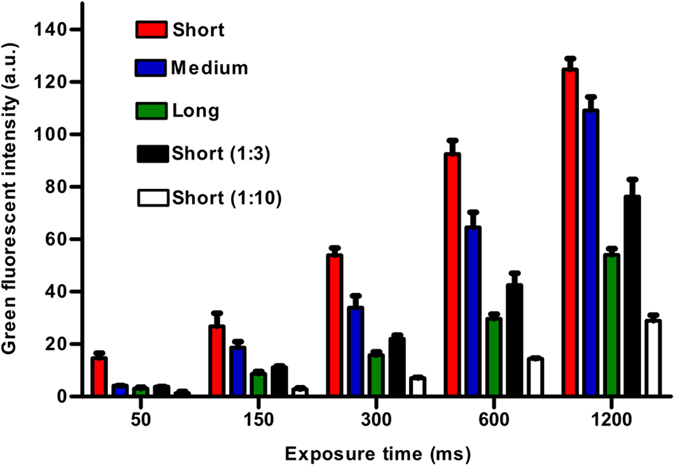
Characterization of the density of FITC-labelled peptide. Short length PEG (short); medium length PEG (Medium); long length PEG (Long); 3 and 10 times non-reactive PEG diluted short length PEG, short (1:3) and Short (1:10), respectively; coupled silicon wafers at 50, 150, 300, 600 and 1200 ms exposure time under 63 x oil lens. Histogram bars represent mean ± s.e.m for three experimental repeats.

It is well known that the density of adhesive proteins such as fibronectin, collagen or fragments containing their adhesive ligands on surfaces can play a major role in determining cellular behaviour^9^. We used ellipsometry to determine the surface density of the three PEG linkers (Table 1). The grafting densities for short:medium:long tethers followed the trend 10:4:1 showing that the ligand density of substrates coated with long linkers was ten times smaller than that of substrates coated with short linkers. Next, we used fluorescein isothiocyanate (FITC) labelled peptide to characterize the density of fluorescent ligands on each of the three surfaces (Fig. 5). This set of data showed a ligand ratio of 3:2:1 for short:medium:long linkers. The measurements from the fluorescence assay possibly underestimated the density of PEG ligands (compared to those obtained by ellipsometry) on the shortest (denser) tether due to local aggregation and quenching.

**Figure 6:**
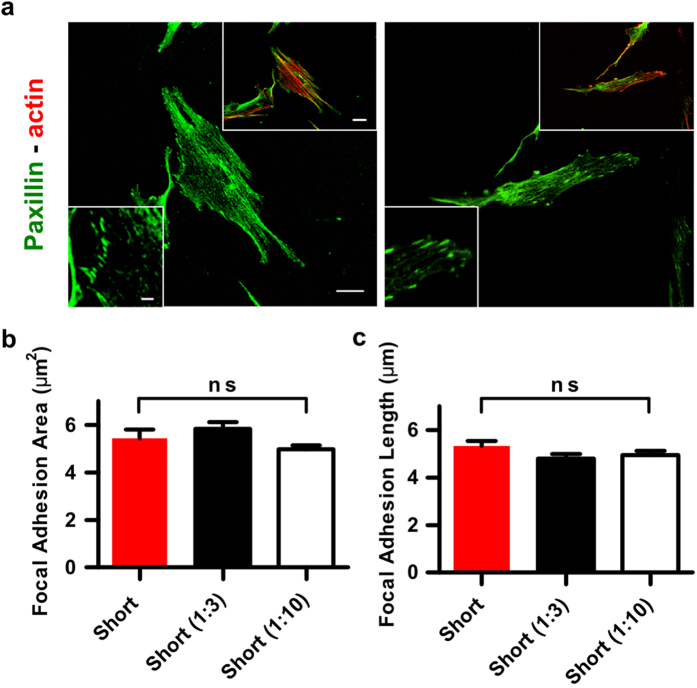
Adhesive ligand tether density does not affect the size and length of focal adhesions. (**a**) Confocal microscopy images of cells stained for paxillin (green) and F-actin (red). Cells were seeded on short linkers diluted with non-reactive short linkers in a ratio 1:3 and 1:10. Scale bars are 20 μm in main image and cell inset and 5 μm in FAs inset. (**b**,**c**) Quantification of focal adhesion area and length (n > 100 for each condition). Histograms represent mean ± sem.

In order to interrogate if this difference in ligand density may account for the differential cellular behaviours we report in this study, cells were seeded on glass surfaces coated with a mixture of the short linkers diluted with non-reactive PEG linkers (same size as short linker) using two different ratios 1:3 and 1:10 (Fig. 6a). There were no significant differences in the length and size of the focal adhesions for cells plated on glass coated with these two ratios or the undiluted short linker (Fig. 6b,c). Likewise, we saw no difference in cell density when cells were seeded on fibronectin, short linkers, or short linkers diluted 1:3 or 1:10 (Supplementary Figure 2).

Our results give direct evidence that the length of cell adhesive tethers, above 40 nm, constitutes a critical sensing distance over which cells integrate mechanical responses. We find that, as well as tether length, the density of ligands plays an important role in mediating such a response, which is very similar to what is observed on matrices with varying mechanical behaviour. However, our experiments give clear evidence that changes in ligand density itself do not account for the observed changes in cellular behaviour reported in this study. A 10-fold dilution of ligands with short tethers (the upper limit within which we have determined the ligand density for long tethers) does not impact cell spreading, density, or focal adhesion formation significantly. Calculations of the grafting densities corresponding to these monolayers indicate that the spacing between two RGD ligands is 1.6 nm for short tethers and 5.1 nm for long tethers. A 10-fold dilution would result in a 3-fold increase in this distance. Hence these values are well within the 60–70 nm critical distance that was identified by Spatz and co-workers^31^,^32^ and is in good agreement with our observation that cells do not respond to ligand density within this range.

### A working model for sensing ECM rigidity and tether length

**Figure 7:**
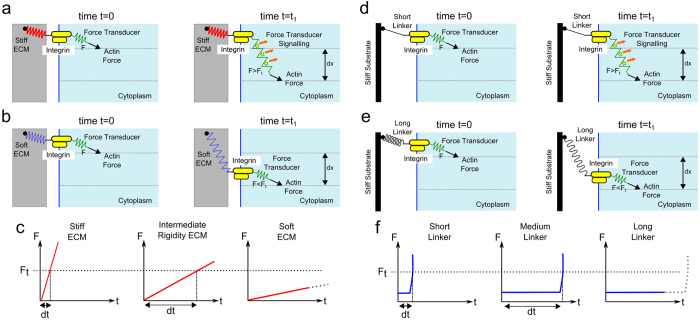
Proposed model to explain both rigidity and tether length sensing. (**a**) The force transducer (FT) molecule (green spring) is coupled such that an ECM-integrin-FT-actin chain exists. On a stiff ECM (red spring) the transmembrane integrin remains at a fixed location whilst the rearward actin flow pulls on the FT. The FT experiences a force greater than the trigger force (F > Ft) which leads to stretching and signalling by exposure of cryptic binding sites. (**b**) On a soft ECM (blue spring) the integrin is unhindered and able to freely diffuse along the membrane as the actin rearward flow applies a force on the FT. The FT experiences a force lower than the trigger force (F < Ft) and therefore signalling does not occur. (**c**) A steadily increasing force profile for an elastic solid is assumed. Differential signalling can be understood by noting the time taken (dt) for the FT to reach the trigger force is less for a stiff substrate than one with intermediate stiffness. Given the periodic nature of the actin rearward flow, for a stiff substrate a greater time will be spent above the trigger force leading to greater signalling. For a soft substrate the FT may never reach the trigger force. (**d**) The integrin is tethered to a stiff substrate (or ECM) by a short flexible tether (black line). This provides tension against integrin movement, analogous to a stiff substrate and leading to FT signalling. (**e**) For a long flexible linker (wavy black line) the integrin is free to diffuse and signalling does not occur, analogous to a soft substrate. (**f**) For a flexible tether a different force profile occurs with a latent period followed by a sharp increase in force. Regardless of force profile, if the FT feels a force greater than the trigger force it will signal. A short linker is thus analogous to a stiff substrate and a long linker is analogous to a soft substrate.

Building on previous work^33^,^34^,^35^, we propose a spatiotemporal mechanism by which cells are able to sense tether length (Fig. 7). We emphasize that this is a preliminary model which in order to be proven would require a direct measurement of the forces acting at the adhesion receptors. A molecular force transducer (FT) is coupled at one end to the actin cytoskeleton and to the cytoplasmic tail of an integrin at the other such that an ECM-integrin-FT-actin chain exists, with actomyosin contraction applying a periodic force with a constant rearward velocity^36^ for a fixed time. With the integrin attached to a stiff substrate, it remains at a fixed location as does the integrin bound end of the FT molecule. Actin rearward flow pulls on the FT molecule at the other end, resulting in the FT molecule experiencing a force greater than a minimum trigger force (F > F_t_). This permits stretching of the FT and possible signalling, for example by exposure of cryptic binding sites (Fig. 7a). With the integrin attached to a soft ECM there is little ECM directed resistance along the chain and the FT molecule experiences a low force (F < F_t_) due to actin flow which is not sufficient to stretch the FT molecule, preventing signalling (Fig. 7b). For a stiff ECM the force on the FT will quickly rise above the trigger force, whereas for a substrate with intermediate rigidity it will take a greater time. Therefore, differential signalling may occur due to the amount of time the FT spends above the trigger force, with increasing time leading to exposure of more cryptic sites, or increased ligation by signalling molecules (Fig. 7c). For integrins attached to stiff substrates with a short tether (Fig. 7d, black line), tension exists which prevents integrin movement, analogous to a stiff substrate and leading to FT signalling. For a long flexible tether (Fig. 7e, wavy black line) the integrin is free to diffuse and signalling does not occur, analogous to a soft substrate. For a flexible tether molecule, the resistance to a pulling force will be small and steady for some time, after which point the linker molecule becomes taught, leading to a sharp increase in resistance force (Fig. 7f). Therefore, for the purposes of signalling the short tether is equivalent to a stiff substrate, and a long tether is equivalent to a soft substrate. For the *in vivo* scenario, it is expected that a combination of tether length and ECM rigidity sensing leads to signalling.

In summary our results suggest that adhesive ligand tether length is another physical cue which cells can sense, in a similar way that cells are known to sense rigidity and protein adhesive ligand density. Although it is unclear whether the extreme range of length scales studied here are physiologically relevant to a cell in its native environment, the experiments provide key insights into understanding the mechanism of cellular mechanosensing. This is illustrated by the tether length-dependent effects on cell surface density and spread area. We see a 75% and a 50% reduction in cell surface density and spread area respectively when comparing the shortest and longest linkers. Importantly, for bioengineering applications the length parameter can be tuned in order to modulate cellular behaviour, independently of matrix mechanics. Hence, depending on the tether length, stiff substrates may be perceived as much softer. By determining the length scale over which cells are able to sense their environment we also provide constraints for those models trying to understand the mechanism of mechanosensing.

## Methods

### RGD Coupled Glass Surface Preparation

Circular glass coverslips with polyethylene glycol tethered RGD ligands were prepared with a three step coupling procedure. The protocol is modified from a procedure described by Popa *et al*.^23^.

1. **Silanization:** Coverlips were cleaned with 1% Hellmanex solution (Sigma-Aldrich) in a sonicator bath for 30 minutes followed by extensive washing. These were then sonicated for 30 mins in acetone, washed and sonicated in ethanol for 30 mins before a final wash in ethanol. They were dried with nitrogen and placed in the oven for 20 mins at 90 °C. After washing, the coverslips were placed in oxygen plasma for 15 minutes. Samples were then immediately silanized using a 1% ethanolic solution of aminopropyltrimethoxysilane (APTMS, Sigma-Aldrich) for 20 minutes followed by extensive washing in ethanol. Samples were then dried with nitrogen and placed in an oven for 30 minutes at 90 °C.
2. **NHS-PEG-Mal coupling:** The short NHS-PEGn-Maleimide crosslinker (1.4 kDa, 9.5 nm) was purchased from Thermoscientific. The medium and long length crosslinker (5.0 kDa, 38.2 nm and 40 kDa, 318 nm respectively) were purchased from NOF Europe GmbH. Upon receipt of the linkers they were dissolved in anhydrous DMSO (Sigma-Aldrich) at 250 mM, 50 mM and 1.6 mM for the short, medium and long linker respectively. They were aliquoted, flash frozen in liquid nitrogen and capped with gaseous nitrogen before storing in a freezer at −80 °C. For the short linker, the stock aliquot was resuspended in borax buffer (20 mM borax, pH 8.5, Sigma-Aldrich). For the medium linker the stock aliquot was resuspended in borax buffer (20 mM borax, pH 7.9. For the long linker, triethylamine (NEt_3_, Sigma-Aldrich) was added to the DMSO stock at a 1:1.5 molar ratio (1.6 mM linker, 2.4 mM NEt3. For each linker, 20 ul was sandwiched between two freshly silanized glass coverslips and the samples stored in a dark, humid chamber for 1 hour. The samples were then washed with copious amounts of distilled water. The nonreactive linker, NHS-PEG (no reactive maleimide group), was purchased from Thermofisher, catalogue number 22687.
3. **RGD peptide coupling:** The cyclo (Arg-Gly-Asp-D-Phe-cys) peptide (RGD) was purchased from Peptides International. It has been shown that cyclic RGD peptides are more stable against enzymatic degradation than linear peptides^37^. The RGD peptide was immediately dissolved in anhydrous DMSO upon receipt at 100 mM, aliquoted, flash frozen in liquid nitrogen and capped with gaseous nitrogen. The aliquots were stored in a freezer at −80 °C. The aliquot was resuspended in buffer (20 mM borax, pH 8.5) to 5 mM and sandwiched between two linker-coupled glass coverslips. Coverslips were allowed to react overnight, after which they were washed extensively with distilled water. The reaction was then quenched by treating surfaces with a 50 mM solution of 2-mercaptoethanol (sigma) for 5 minutes before a final wash in distilled water. Any water on the surfaces was wicked away and samples stored.

### Fibronectin coated glass surfaces

Glass coverslips were coated with 10 ug/ml fibronectin (Sigma-Aldrich, F0895) in PBS for 1 hour at room temperature. Surfaces were then washed with PBS.

### Cell culture and seeding

Human Foreskin Fibroblasts cells (HFF) were grown in DMEM high glucose (Sigma-Aldrich, D6429) with 10% FBS (Sigma-Aldrich, D7524), streptomycin and penicillin (Sigma-Aldrich, P4333) under normal culture conditions (5% CO_2_, 37 °C). Cells were seeded onto the RGD-coupled glass surfaces and the fibronectin (Gibco, PHE0023) coated surfaces in 24-well plates (Corning, 3337) using serum free media (DMEM only) at 37 °C, 5% CO_2_. After 17 hours the samples were washed with PBS (Sigma-Aldrich, PBS1) and media replaced with serum-containing media (DMEM, 10% FBS, S/P). After 4 hours the samples were washed with PBS and fixed using 4% PFA (Sigma-Aldrich, 158127) in PBS for 10 minutes.

### Immunofluorescence staining and microscopy

Fixed HFF cells on glass substrates were permeabilzed with 0.1% Triton-X100 (Sigma-Aldrich, T8787) containing 1% BSA (Sigma-Aldrich, A8022) in PBS for 20 minutes at room temperature. Substrates were then incubated with 0.1% phalloidin (Invitrogen, A22283) containing 1% BSA in PBS for 20 minutes at room temperature. Samples were washed and mounted with ProLong gold antifade reagent (Invitrogen, P36931) and allowed to cure overnight. Epifluorescence images of the cells were obtained with an inverted microscope (Eclipse Ti, Nikon) and a 20x objective using a sCMOS camera.

### AFM characterization of surfaces

The AFM experiments were carried out using a single-molecule AFM developed by the Fernandez Lab at Columbia University and built by Luigs Neumann^23^. A protein construct containing the HaloTag enzyme unit was incubated on the surfaces for 30 minutes. This protein was then extended using OBL-10, gold-coated cantilevers (Bruker) operating at constant retraction rate of 400 nm/s. Traces were then analysed to find the extension at which the force exceeded 42 pN. Data was collected and analysed using Igor Pro software (Wavemetrics).

### Analysis of cell spread area and surface density

A custom routine developed in Matlab was used to aid fast and accurate determination of the cell spread area. Only actin-stained fluorescence images were used for the analysis as the sharp contrast in these images permits easy identification. Images were thresholded and cell perimeters identified. Based on the number of pixels within the cell perimeter, the cell area was calculated. Only cells that were not visibly touching another cell were considered in the analysis as it has been shown that cell-cell contacts can override rigidity and mechanosensing^11^,^17^,^35^. More than 200 cells were averaged for each condition across 4 different substrates for the RGD coupled surfaces and 100 cells across two different substrates for each of the vehicle control samples.

### Ellipsometry

Silicon wafers were cut into 1 cm × 1 cm and followed the treatments as the glass surface for the silanisation and NHS-PEG-Mal coupling for three different PEG length (S-PEG, M-PEG and L-PEG respectively). Non-reactive MS(PEG)24 Methyl-PEG-NHS was used to dilute the short linker to 3 (S(3)-PEG) and 10 times (S(10)-PEG), then coupled to the silicon surface. Dry ellipsometry measurements were used to determine the thickness of PEG monolayers and carried out after each treatment of silicon wafers which were rinsed with deionized water and ethanol and dried under nitrogen flow. For the determination of the grafting density of PEG monolayers, the following equation (Equation S1) provides a relationship between the initial brush thickness h_d0_, the grafting density σ(a value of 1.12 was used for PEG), the molecular weight M_n_ (or degree of polymerisation X_n_ and molar mass of the repeating units M_0_), the density\rho and the Avogadro number N_A_.


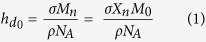


### Characterization of density of FITC labeled RGE

FITC labelled RGE (GCGYGRGESPG-Lys-FITC-G) was diluted in 20 mM, pH 8.5 borax buffer to 5 mM and sandwiched between two linker-coupled silicon wafers overnight in fridge under dark. Then they were rinsed extensively with deionised water. The reaction was then quenched by treating surfaces with a 50 mM solution of 2-mercaptoethanol for 5 minutes before a final wash in deionised water and dried with nitrogen flow. All the samples were then sandwiched with thin cover slips under one drop of deionized water. Images were acquired with a Leica Epifluorescence microscope under 63 x oil lens at different exposure times.

## Additional Information

**How to cite this article**: Attwood, S. J. *et al*. Adhesive ligand tether length affects the size and length of focal adhesions and influences cell spreading and attachment. *Sci. Rep.* **6**, 34334; doi: 10.1038/srep34334 (2016).

## Supplementary Material

Supplementary Information

## Figures and Tables

**Table 1:** Ellipsometry characterisation of PEG coupled silicon wafers reported as grafting density of the PEG chains on each surface.

Sample^a^	M_w_^b^	PEG thickness (nm)	SEM (nm)^c1^	Grafting density σ (chains/nm^2^)^d^	SEM (nm)^c2^	Ratio^e^	Area per chain (nm^2^)^f^	Distance between chains (nm)^g^
S-PEG	1400	0.97	0.10	0.42	0.04	1.0	2.39	1.55
M-PEG	5000	1.30	0.14	0.16	0.02	2.7	6.40	2.53
L-PEG	40000	2.57	0.14	0.04	0.00	10.8	25.90	5.09
S(3)-PEG	1275	0.84	0.07	0.40	0.03	3.0	2.52	1.59
S(10)-PEG	1202	0.72	0.03	0.36	0.02	10.0	2.77	1.66

^a^Abbreviations: S-PEG, short tether; M-PEG, medium tether; L-PEG, long tether; S(3)-PEG/S(10)-PEG: 3 or 10-fold dilution of short tether with non-reactive PEG (MS(PEG)24 Methyl-PEG-NHS). ^b^M_w_: Molecular weight of the PEG tethers used (for mixed monolayers of PEG chains, the molecular weight was calculated from the corresponding weighed molecular weights of the two tethers according to *Mw* (*mixed monolayers*) = *Mw*(*S*−*PEG*)/*dilution times* + (*dilution times* −1) *Mw*(*non*-*reactive PEG*)/*dilution times*. ^c1,c2^*SEM: standard error of the mean for three repeats.*^d^Grafting density: determined using Equation 1 in methods. ^e^Ratio: determined from the ratios of corresponding calculated grafting densities with respect to the grafting density of the shortest tethers. ^f^Area per chain: 1/σ. ^g^Distance between chains: square root of f.
