# Supplementary material for: Adhesive ligand tether length affects the size and length of focal adhesions and influences cell spreading and attachment
Source: Sci Rep. 2016 Sep 30;6:34334. doi: 10.1038/srep34334 (PMC5043376; doi:10.1038/srep34334)
Supplement: Supplementary Information [file srep34334-s1.pdf]

Supplementary figures

Adhesive ligand tether length affects the size and length of focal adhesions and influences cell spreading and attachment

Simon J. Attwood[1], Ernesto Cortes[1], Alexander W. M. Haining[1], Benjamin Robinson[1], Danyang Li[2,3], Julien Gautrot[2,3], & Armando del Río Hernández[1§]

[1] Cellular and Molecular Biomechanical laboratory, Department of Bioengineering, Imperial College London, United Kingdom

[2] Institute of Bioengineering and [3] School of Engineering and Materials Science, Queen Mary, University of London, Mile End Road, London, E1 4NS, UK.

[§]Corresponding author:

Armando del Río-Hernández, PhD
Cellular and Molecular Biomechanics Laboratory http://biomechanicalregulation-lab.org/ Department of Bioengineering
Imperial College London
London SW7 2AZ, UK
email: a.del-rio-hernandez@imperial.ac.uk
telephone: +44(0) 2075 945 187
Mobile: +44(0)7407270946

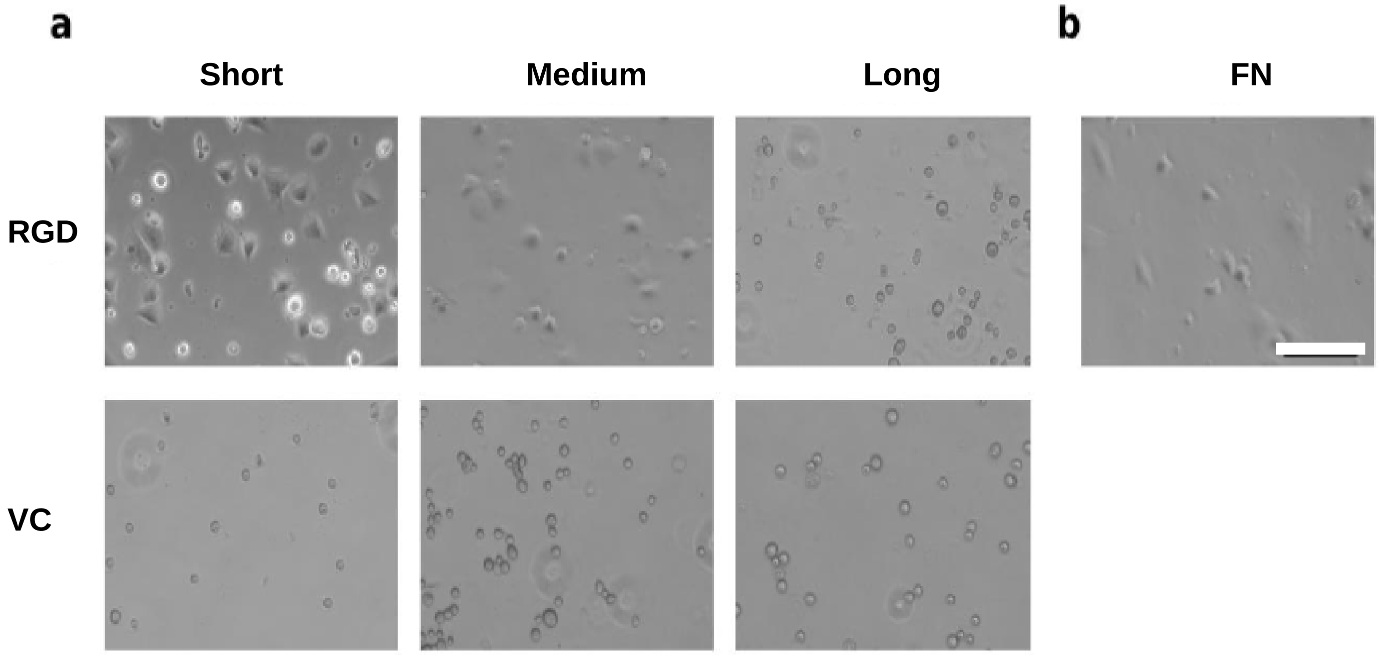

Supplementary figure 1| Differences in cell behaviour are observed 2 hours after seeding. a, Upper row: Phase contrast microscopy of cells on RGD-coupled surfaces prepared with short (9.5nm), medium (38.2nm) and long (318nm) polyethylene glycol (PEG) tethers. Cell spread area decreases with increasing tether length. No effects on cell surface density are seen as surfaces have not yet been washed; Lower row: Vehicle control (VC) surfaces with short, medium and long length tethers but lacking the RGD adhesive ligand. All cells appear small and rounded. **b,** Cells on FN coated glass exhibit similar spread area as compared to cells on RGD surfaces with the shortest tethers. Scale bar is 150μm

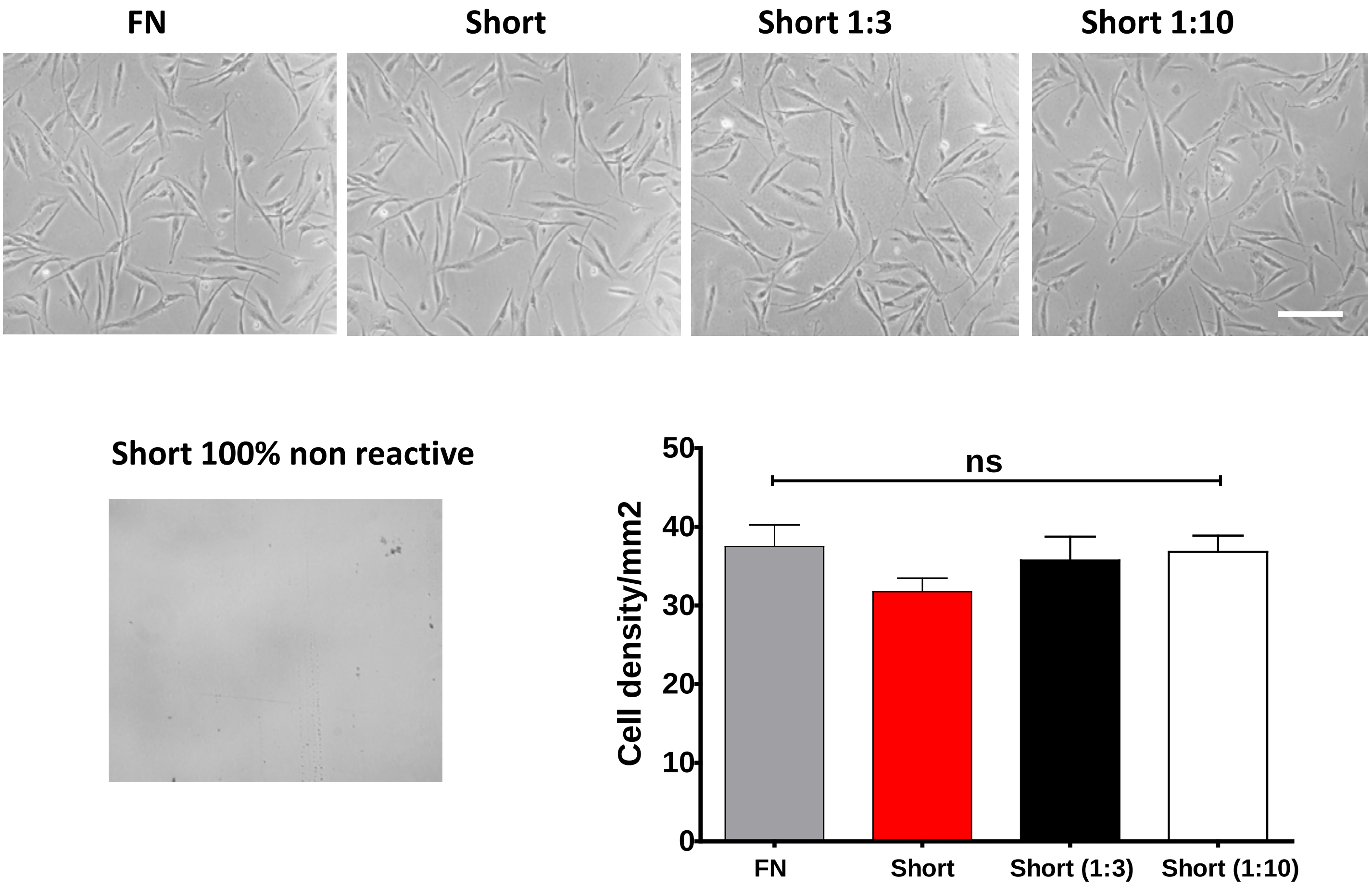

Supplementary figure 2| Adhesive ligand tether density does not affects cell density. Top: Bright field images of cells plated on glass coated with fibronectin (FN), short linkers (short), and short linkers diluted with non reactive linkers in the ratio 1:3 and 1:10, respectively. Scale bar 200 μm. **Bottom**: Representative bright field image of glass coated with non reactive short linkers and seeded with cells. Cell attachment was neglectable. The graph shows the quantification of cell density (n>3). Histograms represents mean ± sem. One way ANOVA and Tukey's post hoc test. ns, non significant
